# Healthy immigration effect among internal migrants in megacities: a cross-sectional study in Shanghai, China

**DOI:** 10.3389/fpubh.2023.1167697

**Published:** 2023-06-12

**Authors:** Enhong Dong, Ting Xu, Jiahua Shi, Dongjiao Ba, Haiwang Zhou, Zhijian Li, Cheng Huang

**Affiliations:** ^1^School of Nursing and Health Management, Shanghai University of Medicine and Health Science, Shanghai, China; ^2^School of Media and Communication, Shanghai Jiao Tong University, Shanghai, China; ^3^Institute of Healthy Yangtze River Delta, Shanghai Jiao Tong University, Shanghai, China; ^4^Huangpu District Health Promotion Center, Shanghai, China; ^5^XinHong Community Health Service Center of Minhang District, Shanghai, China; ^6^Institute of Urban and Demographic Studies, Shanghai Academy of Social Sciences, Shanghai, China; ^7^Department of Human Resources Management, Shanghai Sixth People’s Hospital, Shanghai, China; ^8^Antai College of Economics and Management, Shanghai Jiao Tong University, Shanghai, China; ^9^The Center for Health Economics and Management, Shanghai Jiao Tong University, Shanghai, China

**Keywords:** internal migrant, healthy immigration effect, determinants, self-rated health, megacity

## Abstract

**Objectives:**

To verify the healthy immigration effect on self-rated health (SRH) among Chinese internal migrants, identify the determinants of SRH, and provide recommendations for the Chinese government to formulate effective intervention strategies to improve population governance and health management in megacities.

**Methods:**

A sample comprising 1,147 white-and blue-collar migrant workers was randomly selected through an online survey conducted in Shanghai from August to December 2021. Multivariate logistic regression models were used to verify the healthy immigration effect as well as determinants of the effect among internal migrants in Shanghai.

**Results:**

Among 1,024 eligible internal migrants, 864 (84.4%) were aged between 18 and 59 years, 545 (53.2%) were men, and 818 (79.9%) were married. When confounders in the logistic regression models were adjusted, the odds ratio of SRH for internal migrants who had lived in Shanghai for 5–10 years was 2.418 (*p* < 0.001), whereas the odds ratio for those who had lived there for ≥10 years was not statistically significant. Additionally, marital status, a postgraduate or higher degree, income level, number of physical examinations in the past 12 months, and the number of critical diseases they were suffering from, were significant contributing factors for favorable SRH among the internal migrants. Furthermore, a cross-sectional analysis revealed that SRH demonstrated a healthy immigration effect among blue-collar internal migrants from the manufacturing industry but not among white-collar internal migrants.

**Conclusion:**

A healthy immigration effect was observed among internal migrants in Shanghai. The migrant population that had lived in Shanghai for 5–10 years had more health advantages than the locals, whereas those who had lived there for ≥10 years did not. The Chinese government should understand this effect and enact measures accordingly, such as implementing physical examinations, improving acculturation, addressing individual characteristics, and improving socioeconomic conditions to improve the physical and mental health of internal migrants. Enacting such changes could facilitate the integration of migrants into the local culture of megacities.

## 1. Introduction

With the developing economy and accelerating urbanization, China’s aging population is increasing rapidly. For example, according to statistics from the seventh national population census in 2021, the proportion of Shanghai’s population over the age of 60 years was 23.38%, and that over the age of 65 years was 16.28%. Therefore, Chinese cities, especially megacities, urgently require internal migrants to alleviate the so-called brain drain. Because of this need, the migrant population in megacities has gradually increased in recent years. In 2018, approximately 355 million internal migrants were recorded in the four first-tier megacities of Beijing, Shanghai, Guangzhou, and Shenzhen (1). In Shanghai alone, approximately 9.7 million migrants were recorded, accounting for 40.13% of all permanent residents.

Shanghai, the biggest megacity in China, has set a target to become a global technological innovation center. To counter population aging and brain drain, the city needs to attract more internal migrants, especially high-tech talent, to increase the human capital. Because internal migrants contribute to urban development, they must be integrated into the city both economically and culturally. According to the healthy immigrant effect (HIE) theory, also known as the epidemiological paradox, migrants tend to have more health advantages than locals. Jasso et al. (2) evaluated data from the United States and determined that migrants, especially those who have resided in the country for <10 years, have evident health advantages compared with native residents. In addition, McDonald and Kennedy (3) analyzed Canadian immigration data and determined that even after controlling for countries of origin, the migrants exhibited better physical health compared with locals. However, institutional barriers, such as the inability to share basic public services with locals, poor access to medical care and medical insurance, and a lack of self-health awareness, can increase health risks among internal migrants in megacities. Certain infectious diseases, chronic diseases, and mental health conditions are more prevalent among internal migrants (4–7). Furthermore, the migratory patterns of internal migrants affect the health of other groups (8), which in turn affects governance, regional development, societal stability, and economic equality, especially in Chinese megacities.

Therefore, with respect to the Chinese population flow, verifying the presence of the HIE is essential. Related studies have focused primarily on HIE among international migrants between countries (9–12). Moreover, there have been some studies on the health effects of internal migration. For example, Wen et al. (13) explored the neighborhood effects on self-rated health (SRH), chronic conditions, and psychological well-being of internal migrants and urban natives in Shanghai. Chen (8) examined the potential associations between migration path and health outcomes among internal migrants in Beijing. However, to the best of our knowledge, research focused on the identification of HIE by examining the association between years of residency and health outcomes among highly educated and skilled internal migrants in the megacities of China, has never been reported. Thus, to fill the research gap, we analyzed data from Shanghai to verify the HIE of internal migration in China, investigated the different trajectories of residency time on migrants’ SRH, and identified the determinants of migrants’ SRH. The results could provide a reference for the local government to explore creative health-management policies, and improve the quality of social governance in other Chinese cities as well as in other countries in cases of shared similarities.

## 2. Materials and methods

### 2.1. Participants and study design

A sample comprising 1,147 white-and blue-collar migrant workers was randomly selected from manufacturing enterprises in Shanghai through a questionnaire posted on the online survey platform “Star,” between August and December 2021. Considering the disadvantages of online surveys (such as sampling issues, access to a unique population, and selection bias in selecting the study samples) we practiced the following four methods to ensure unbiased representation of the research population and quality of the data:

Method 1: The questionnaire set the first filling threshold and the single IP restriction response method. For example, participants were asked whether they were internal migrants or not and whether they had lived in Shanghai for >6 months. Participants replying in the negative would finish the survey, and would be excluded from the study.

Method 2: A respondent-driven sampling technique was applied to collect data. Respondent-driven sampling combines the snowball sampling method with the method of assigning weights to samples. This type of sampling incentivizes respondents (also called “seeds”) to recommend a specific number of peer groups, thereby reducing sampling bias through multiple recommendations of seeds.

Method 3: To encourage participants to actively fill in the questionnaire, electronic WeChat red envelopes (a form of cash bonus) were issued immediately after survey completion, to improve the response rate and control the data quality simultaneously.

Method 4: As the data collection progressed, a dynamic monitoring method was adopted to consistently identify groups that did not meet the conditions, or those whose proportion was too high even though they did meet the conditions, so that the questionnaire distribution channels could be adjusted timely and accordingly.

The online questionnaire consisted of 47 questions that were required to be answered using a single IP address. Respondents were excluded if (i) they were under the age of 18 years, (ii) completed the survey in <200 s, (iii) provided duplicate answers or followed similar answer patterns, (iv) missed 5% or more values, or (v) provided answers with logical errors.

Due to the possibility of information leaks, the respondents’ real names, contact details, and other private information was not sought. The questionnaire for this study was based on a literature review and contained variables such as (i) demographic and socioeconomic characteristics (for example, age, sex, marital status, occupation, insurance cover, income, number of years of residence, and place of birth); (ii) use of health-care options (that is, frequency of physical examinations and physician visits); and (iii) health-related profile, including health status (for example, self-rated physical, mental, and social health status), and numbers of chronic diseases (disease І) or critical diseases (disease II) they are currently suffering from.

This study was conducted in compliance with the Shanghai University of Medicine and Health Sciences Institutional Review Board for the Protection of Human Subjects (No. 2019-gskyb-02-372,424,198,012,222,511; March 5, 2019). All participants provided written informed consent.

### 2.2. Measures

Based on expert consultations and data derived from previous literature (14–16), the duration of residence was divided into the following four stages: 6 months–1 year, 1–5 years, 5–10 years, and > 10 years.

Health-care use was measured based on the frequency of physical examinations and physician visits in the past 12 months, and the preferred health-care institution. Frequencies were answered using a 4-point Likert scale (1 = none, 2 = once, 3 = two times, 4 = three times or more); higher the score, more frequently had the participant used health-care services. The preferred type of health-care institution was dichotomized into hospitals and primary care institutions.

Health status was measured using a revised version of the 12-item Short Form Survey that was developed previously (17) and consisted of five items, including those related to physical health, mental health, and social support. Each item was anchored with endpoints at 1 (excellent) and 5 (poor). A lower score indicated a more favorable SRH status. We collapsed the five categories to form a dichotomous measure: excellent/very good/good (favorable) vs. fair/poor (not favorable). In this study, the internal consistency coefficient of the revised Short Form Survey was set at 0.8312.

“Disease І” consisted of ten common chronic health conditions, namely asthma, back pain, hypertension, hyperlipidemia, diabetes, allergies, migraine, ulcers, bronchial inflammation, and arthritis (3). These conditions are normally not considered to be life-threatening. The number of “disease І” conditions the participant had was determined using a 3-point Likert scale (0 = none of the above, 1 = one, and 2 = two or more). “Disease II” encompassed three common critical diseases, namely, heart disease, blood-related cancers (such as leukemia), and other cancers. The number of “disease II” conditions was determined using a 2-point Likert scale (0 = none of the above and 1 = one or more). The classifications and measures for scoring variables are shown in Table 1.

**Table 1:** Classifications and measures of scoring variables.

Variables	How to measure	Type
SRH	1 = favorable; 0 = not favorable	Categorical
Years of residency	1 = 6 months–1 year; 2 = 1–5 years; 3 = 5–10 years; 4 = ≥10 years	Categorical
Age	1 = ≤39 old years; 2 = ≥40–60 old years	Categorical
Gender	0 = Female; 1 = Male	Categorical
Marital status	0 = No; 1 = Yes	Categorical
Educational level	1 = high school or below; 2 = University (including junior college); 3 = graduate or above	Categorical
Industry type	1 = White-collar; 2 = Blue collar	Categorical
Insured type	1 = UEBMI; 2 = URRBMI; 3 = FI; 4 = CMI or others	Categorical
Annual income	1 = CNY100,000 or below; 2 = CNY110,000–CNY250,000; 3 = CNY260,000–CNY400,000; 4 = CNY410,000–CNY600,000; 5 = CNY600,000 or more	Categorical
The frequency of physical examinations in the past year	1 = None; 2 = once; 3 = Two times; 4 = Three times or more	Categorical
The frequency of physician visits physician in the past year	1 = None; 2 = once; 3 = Two times; 4 = Three times or more	Categorical
The preferred health-care institution	1 = Hospitals; 2 = Primary care institutions	Categorical
The number of disease І you have	0 = None of the above; 2 = One; 2 = Two or more	Categorical
The number of disease II you have	0 = None of the above; 1 = One or more	Categorical

SRH, self-rated health; UEBMI, urban employee basic medical insurance; URRBMI, urban and rural residents basic medical insurance; FI, free insurance; CMI, commercial medical insurance.

### 2.3. Procedure

The Kolmogorov–Smirnov (K–S) test was conducted to test the normality of the data and was followed by a descriptive analysis of the demographic and socioeconomic characteristics, health-care use, and disease types. We then conducted an independent sample *t*-test and one-way analysis of variance (ANOVA) to identify statistically significant differences between the study groups. Additionally, Pearson’s correlation analysis was performed to test linear relationships between continuous variables. Statistically significant covariates from the one-way ANOVA and *t*-tests were retained for subsequent multivariate logistic regression analysis.

To determine the HIE, we verified whether an internal migrant participating in the study had a more favorable SRH than a local resident. Because age (18), sex (19), marital status (20), educational level (3), income (21, 22), health-care use (23), and the numbers of “diseases І and II” (24) also affect SRH, we set these variables as confounding factors in models 2, 3, and 4 (model 1 was an unadjusted null model). In model 2, we adjusted for demographic and socioeconomic characteristics. In model 3, we adjusted for health-care use based on the data derived using model 2. In model 4, we included the numbers of “diseases І and II” based on the data derived using model 3. To identify multicollinearity among the independent variables, we used the variance inflation factor index. If the variance inflation factor score was lower than “5,” the variable was considered acceptable, and included in the multivariate regression analysis.

To assess whether a predictor had a confounding effect, a difference of 20% or more in the coefficients of number of years of residence was used as the cut-off point. Effect modifications were investigated by examining all possible two-way interactions for the main variable of interest, and including predictors and confounders in the main effect model. Each interaction was assessed according to its *p*-value and Akaike information criterion for subsequent comparisons. To identify the goodness of fit and predictability of the model, we used the receiver operating characteristic curve and area under the receiver operating characteristic curve (AUC) index. The results are presented as adjusted odds ratios (ORs).

### 2.4. Statistical analyses

All statistical analyses were conducted using Stata, version 15.0 (StataCorp, College Station, TX, United States). A two-tailed *p*-value < 0.05 indicated statistical significance.

## 3. Results

### 3.1. Descriptive characteristics

In this study, 1,024 eligible respondents were selected from 1,147 total respondents, indicating a valid response rate of 89.28%. Most (84.4%) of the respondents were 18–39 years old, 545 (53.2%) were men, and 818 (79.9%) were married. A total of 827 (80.8%) respondents were university-educated, and 761 (74.3%) had employee medical insurance. Regarding annual income distribution, most (*n* = 503, 49.1%) of the respondents earned between CNY 110,000 and CNY 250,000 (Table 2). To confirm whether the sample could accurately reflect the characteristics of the total migrant group in Shanghai, we made a comparison of the demographic variables between the study sample and the official data of the Shanghai population; the similar data distribution on demographic profile reflected a good representation of our dataset (Supplementary Table S1).

**Table 2:** Results of the descriptive and univariate analyses (*n* = 1,024).

Variables	*N* (%)	SRH		*t*/χ^2^ value	*p* value
		Favorable (%)	Not favorable (%)		
**Age**				5.74	0.017
≤39 old years	864 (84.4%)	138 (16.0%)	726 (84.0%)		
40–60 old years	160 (15.6%)	38 (23.8%)	122 (76.2%)		
**Gender**				3.74	0.154
Female	475 (46.4%)	76 (16%)	399 (84%)		
Male	549 (53.6%)	102 (18.6%)	447 (81.4%)		
**Marital status**				30.20	0.000
No	206 (20.1%)	62 (30.1%)	144 (69.9%)		
Yes	818 (79.9%)	114 (13.9%)	704 (86.1%)		
**Years of residency**				17.69	0.001
<1 year	359 (35.1%)	126 (35.1.6%)	233 (64.9%)		
1–5 years	142 (13.9%)	9 (6.3%)	133 (93.7%)		
5–10 years	153 (14.9%)	28 (18.3%)	125 (81.7%)		
≥10 years	370 (36.1%)	77 (20.8%)	293 (79.2%)		
**Educational level**				24.95	0.000
High school or below	53 (5.2%)	23 (42.3%)	30 (57.7%)		
University (including junior college)	827 (80.8%)	133 (16.1%)	694 (83.9%)		
Graduate or above	144 (14.0%)	20 (13.9%)	124 (86.1%)		
**Insured type**				19.02	0.000
White-collar	487 (47.56%)	377 (77.41%)	110 (22.59%)		
Blue-collar	537 (52.44)	471 (87.71%)	66 (12.29%)		
**Insured type**				6.28	0.099
UEBMI	761 (74.3%)	126 (16.6%)	635 (83.4%)		
URRMI	116 (111.3%)	18 (15.5%)	98 (84.5%)		
FI	30 (2.9%)	3 (10%)	27 (90%)		
CMI	117 (11.4%)	29 (24.8%)	88 (75.2%)		
**Annual income**				38.74	0.000
CNY100,000 or below	198 (19.3%)	63 (31.8%)	135 (68.2%)		
CNY110,000–CNY250,000	503 (49.1%)	66 (13.1%)	437 (86.9%)		
CNY260,000–CNY400,000	243 (23.7%)	35 (14.4%)	208 (85.6%)		
CNY410,000–CNY600,000	47 (4.6%)	5 (10.6%)	42 (89.4%)		
CNY600,000 or more	33 (3.2%)	7 (21.2%)	26 (78.8%)		
**The frequency of physician visits physician in the past year**					
None	142 (13.9%)	33 (23.2%)	109 (76.8%)	8.43	0.038
Once	355 (34.7%)	49 (13.8%)	306 (86.2%)		
Two time	312 (30.5%)	50 (16%)	262 (84%)		
Three times or more	215 (21%)	44 (20.5%)	171 (79.5%)		
**The frequency of physical examinations in the past year**				29.09	0.000
None	135 (13.2%)	45 (33.3%)	90 (66.7%)		
Once	660 (64.5%)	101 (15.3%)	559 (84.7%)		
Two time	186 (18.2%)	24 (12.9%)	162 (87.1%)		
Three times or more	43 (4.2%)	6 (14%)	37 (86%)		
**The preferred health-care institution**				0.93	0.335
Hospitals	745 (72.8%)	119 (16.0%)	626 (84.0%)		
Primary care institutions	279 (27.2%)	91 (32.6%)	188 (67.4%)		
**The number of disease І you have** ^**a**^				9.10	0.011
None of above	578 (56.4%)	89 (15.4%)	489 (84.6%)		
One	371 (36.2%)	65 (17.5%)	306 (82.5%)		
Two or more	75 (7.3%)	22 (29.3%)	53 (70.7%)		
**The number of disease II you have** ^**a**^				53.52	0.000
None of above	955 (93.3%)	142 (14.9%)	813 (85.1%)		
One or more	69 (6.7%)	34 (49.3%)	35 (50.7%)		

a Disease І includes 10 common chronic health conditions, namely asthma, back pain, hypertension, hyperlipidemia, diabetes, allergies, migraine, ulcers, bronchial inflammation, and arthrit; Disease II encompassed three common critical diseases: heart disease, blood-related cancers (e.g., leukemia), and other cancers. SRH, self-rated health; UEBMI, urban employee basic medical.

### 3.2. Univariate analysis and correlation analyses

According to the results of the K–S test, the data of all the dependent and independent variables were acceptable; the skewness values of all variables were near 0, and all the kurtosis values were <3. Furthermore, ANOVA and *t*-tests demonstrated that the variables significantly associated with SRH were age (*t* = 5.74, *p* = 0.017), marital status (*χ*^2^ = 30.20, *p* < 0.001), number of years of residence (*χ*^2^ = 17.69, *p* < 0.001), educational level (*χ*^2^ = 24.95, *p* < 0.001), industry type (*χ*^2^ = 19.02, *p* = 0.001), annual income (*χ*^2^ = 38.74, *p* < 0.001), number of physician visits in the past 12 months (*χ*^2^ = 8.43, *p* = 0.038), number of physical examinations in the past 12 months (*χ*^2^ = 29.09, *p* < 0.001), number of “disease І” (*χ*^2^ = 9.10, *p* = 0.011), and number of “disease II” (*χ*^2^ = 53.52, *p* < 0.001) (Table 2).

### 3.3. Multivariate logistic regression analysis

According to the results of the multicollinearity test, the variance inflation factor index score of each significant independent variable in the multivariate logistic regression model was found to be between 1.06 and 1.29, indicating no multicollinearity.

According to the results of the unadjusted model 1, the OR of <10 years of residency was 3.884 (*p* < 0.001) (Table 3). Similarly, after adjustment for the significant covariates of age, marital status, educational level, industry type, and annual income in model 2, the OR of <10 years of residency was 2.891 (*p* < 0.001). Therefore, regarding SRH, internal migrants who had lived in Shanghai for <10 years were 1.891 times more likely to report favorable health compared with locals. However, the OR of SRH for internal migrants who had lived in Shanghai for ≥10 years was not significant. Additionally, among the statistically significant covariates, the OR of favorable SRH among those aged 40–60 years was 0.642 (*p* < 0.05), which indicated that the OR of favorable SRH in internal migrants decreases with age. Further, internal migrants in Shanghai who were married were 2.103 times more likely to report favorable SRH than unmarried migrants (*p* < 0.001). The ORs of obtaining an undergraduate or a postgraduate (or higher) degree were 2.283 (*p* < 0.01) and 2.797 (*p* < 0.01), respectively, which indicated that better-educated internal migrants in Shanghai were more likely to report favorable health. Similarly, those with an annual income between CNY 110,000 and CNY 250,000 were 1.989 times (*p* < 0.001) more likely to report favorable health compared with those with an annual income of CNY 100,000 or less.

**Table 3:** Results of the multivariate logistic regression analysis (*n* = 1,024).

Independent variables	Model1^a^	Model2^b^	Model3^c^	Model4^d^
**Years of residency**				
Shanghainese	1.000	1.000	1.000	1.000
<1 years	0.885	1.130	1.094	0.938
	(0.500–1.566)	(0.587–2.176)	(0.556–2.154)	(0.459–1.917)
1–5 years	1.424	1.196	1.131	0.983
	(0.944–2.148)	(0.755–1.897)	(0.710–1.801)	(0.609–1.586)
5–10 years	3.884***	2.891***	2.684**	2.418**
	(1.889–7.983)	(1.382–6.046)	(1.266–5.694)	(1.134–5.154)
≥10 years	1.173	1.041	1.007	0.935
	(0.725–1.897)	(0.608–1.780)	(0.583–1.738)	(0.525–1.665)
**Age**				
≤39 old years		1.000	1.000	1.000
40–60 old years		0.642	0.659	0.673
		(0.392–1.053)	(0.392–1.107)	(0.391–1.160)
**Marital status**				
No		1.000	1.000	1.000
Yes		2.103***	1.943***	2.113***
		(1.377–3.212)	(1.245–3.033)	(1.342–3.326)
**Educational level**				
High school or below		1.000	1.000	1.000
University (including junior college)		2.283**	2.291**	1.822
		(1.122–4.648)	(1.129–4.651)	(0.888–3.741)
Graduate or above		2.797**	2.834**	2.578**
		(1.140–6.867)	(1.144–7.025)	(1.011–6.574)
**Insured type**				
White-collar		1.000	1.000	1.000
Blue-collar		1.415	1.349	1.307
		(0.937–2.137)	(0.883–2.061)	(0.848–2.014)
**Annual income**				
CNY100,000 or below		1.000	1.000	1.000
CNY110,000–CNY250,000		1.989***	1.765**	1.840**
		(1.238–3.194)	(1.079–2.888)	(1.111–3.047)
CNY260,000–CNY400,000		1.573	1.366	1.522
		(0.897–2.759)	(0.754–2.474)	(0.824–2.812)
CNY410,000––CNY600,000		1.811	1.514	1.654
		(0.627–5.230)	(0.502–4.569)	(0.514–5.323)
CNY600,000 or more		1.096	0.909	0.984
		(0.421–2.853)	(0.322–2.566)	(0.324–2.982)
**The frequency of physical examinations in the past year**				
None			1.000	1.000
Once			1.971***	2.027***
			(1.185–3.278)	(1.212–3.389)
Two time			2.092**	2.326**
			(1.073–4.079)	(1.190–4.548)
Three times or more			2.514*	2.662*
			(0.869–7.274)	(0.948–7.476)
**The frequency of physical examinations in the past year**				
None			1.000	1.000
Once			1.114	1.182
			(0.612–2.029)	(0.649–2.152)
Two time			0.779	0.899
			(0.433–1.401)	(0.498–1.623)
Three times or more			0.765	0.939
			(0.415–1.409)	(0.496–1.778)
**The number of disease І** ^**e**^**you have**				
None of above				1.000
One				0.872
				(0.568–1.339)
Two or more				0.753
				(0.366–1.550)
**The number of disease II** ^**f**^**you have**				
None of above				1.000
One or more				0.176***
				(0.0991–0.314)
Constant	3.805***	0.615	0.435	0.622
	(2.960–4.891)	(0.127–2.985)	(0.073–2.583)	(0.125–3.103)
Pseudo-*R*^2^	0.0217	0.0755	0.0872	0.1313
Observations	1,024	985		985

a Unadjusted crude model.

b Multivariate model adjusted for demographic and socioeconomic characteristics.

c Multivariate model adjusted for the frequency of physical examinations in the past year, the frequency of physical examinations in the past year as well as demographic and socioeconomic characteristics.

d Multivariate model adjusted for the frequency of physical examinations in the past year, the frequency of physical examinations in the past year, the number of disease І and II one have as well as socio-demographics.

e Disease І includes 10 common chronic health conditions, namely asthma, back pain, hypertension, hyperlipidemia, diabetes, allergies, migraine, ulcers, bronchial inflammation, and arthrit.

f Disease II encompassed three common critical diseases: heart disease, blood-related cancers (e.g., leukemia), and other cancers. SRH, self-rated health; UEBMI, urban employee basic medical insurance; URRBMI: urban and rural residents basic medical insurance; FI, free insurance; CMI: commercial medical insurance.

*p < 0.05; **p < 0.01; ***p < 0.001.

In model 3, when the demographic and socioeconomic covariates, and health-care use were controlled, the OR of living in Shanghai for <10 years was 2.684 (*p* < 0.001), indicating that internal migrants who had lived in Shanghai for <10 years were 1.684 times more likely to report favorable health than Shanghai locals. Similar to the case in model 1, the OR of living in Shanghai for ≥10 years was not significant. Furthermore, the ORs of favorable SRH were 1.971 (*p* < 0.001), 2.092 (*p* < 0.01), and 2.514 (*p* < 0.05) for internal migrants who had undergone one, two, and three physical examinations, respectively, in the past 12 months. These results indicate that favorable SRH increases with the number of physical examinations undergone by the internal migrants.

In model 4, as in model 3, when the covariate of the number of diseases was controlled, the OR of <10 years of residency was 2.418 (*p* < 0.001), indicating that internal migrants who had lived in Shanghai for <10 years were 1.418 times more likely to report favorable health than Shanghai locals. However, the OR of living in Shanghai for ≥10 years was not statistically significant (*p* > 0.10). Additionally, the OR of favorable SRH among the respondents with one or more critical diseases was 82.4% lower than that of those with no critical diseases (OR = 0.176, *p* < 0.001). Regarding the adjusted covariates in models 2 and 3, marital status, education at graduate level or above, annual income between CNY 110,000 and CNY 250,000, and number of physical examinations in the past 12 months were all significantly associated with a favorable SRH.

**Figure 1:**
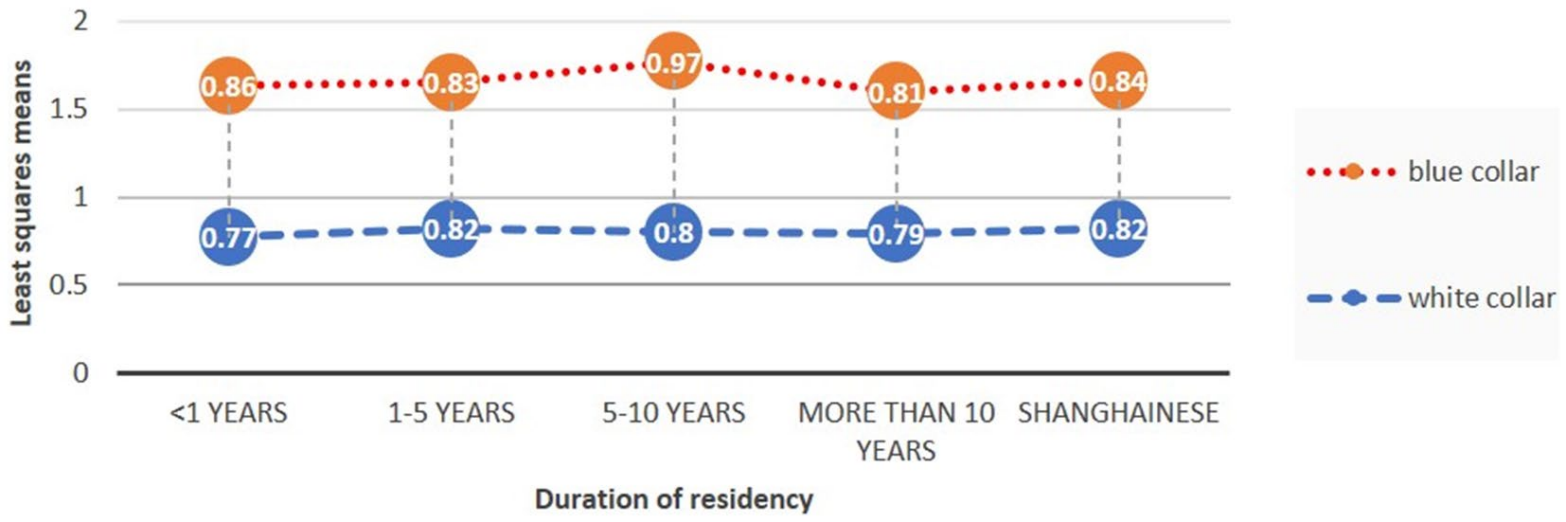
Least squares means (estimates of the linear predictors on the logit scale) and the 95% confidence intervals of SRH for Shanghainese and internal migrants with different durations of residency. SRH, self-rated health.

After examining all possible two-way interactions for years of residency and including predictors and confounders in the main effect model, we found that only the industry type was statistically significant. Therefore, considering the crossover effect of industry type on years of residency, we conducted a logistic regression analysis that applied stratification. The results revealed that the HIE occurred only among blue-collar workers with 5–10 years of residency (OR = 7.068, *p* < 0.012), indicating that blue-collar internal migrants were 7.068 times more likely to report favorable SRH compared with locals. Conversely, no HIE was observed among white-collar migrant workers, which indicated that such workers did not benefit from Shanghai residency in a similar manner (Figure 1 and Table 4). Moreover, we also tested HIE among different groups based on age, sex, and education levels, and found no existing HIE (Table 4).

**Table 4:** Comparison of HIE among internal migrants in different industries in Shanghai, China (*n* = 1,024).

Industry type	AOR	95% CI		*p* value
White collar		Lower	Upper	
Shanghainese	1.000			
<1 year	AOR 0.732	0.257	2.083	0.558
1–5 years	1.092	0.507	2.357	0.821
5–10 years	0.795	0.289	2.189	0.657
≥10 years	1.043	0.456	2.389	0.920
**Blue collar**				
Shanghainese	1.000			
<1 year	1.263	0.460	3.466	0.650
1–5 years	0.918	0.454	1.857	0.812
5–10 years	7.068	1.526	32.735	0.012
≥10 years	0.882	0.362	2.146	0.782
**Gender**				
**Male**				
Shanghainese	1.000			
<1 year	1.028	0.341	3.100	0.961
1–5 years	0.973	0.467	2.031	0.943
5–10 years	1.844	0.576	5.896	0.302
≥10 years	0.602	0.261	1.389	0.234
**Female**				
Shanghainese	1.000			
<1 year	0.787	0.280	2.215	0.650
1–5 years	0.805	0.408	1.588	0.531
5–10 years	3.125	0.070	9.126	0.057
≥10 years	1.016	0.468	2.209	0.967
**Age**				
**≤39 old years**				
Shanghainese	1.000			
<1 year	0.770	0.348	1.704	0.519
1–5 years	0.834	0.518	0.480	0.518
5–10 years	1.844	0.147	0.806	0.147
≥10 years	0.602	0.145	0.305	0.145
**40–60 old years**				
Shanghainese	1.000			
<1 year	2.512	0.349	18.099	0.361
1–5 years	0.523	0.087	3.137	0.478
5–10 years	7.809	0.494	123.566	0.145
≥10 years	1.662	0.591	4.674	0.335
**Educational level**				
**High school or below**				
Shanghainese	1.000			
<1 year	0.601	0.007	1.281	0.820
1–5 years	0.540	0.006	1.095	0.104
5–10 years	0.670	0.007	1.194	0.122
≥10 years	0.550	0.008	1.089	0.123
**University (including junior college)**				
Shanghainese	1.000			
<1 year	0.948	0.422	2.130	0.897
1–5 years	0.962	0.564	1.642	0.888
5–10 years	2.137	0.890	5.132	0.089
≥10 years	1.157	0.549	2.438	0.701
**Graduate or above**				
Shanghainese	1.000			
<1 year	0.934	0.522	2.110	0.897
1–5 years	1.933	0.332	11.259	0.464
5–10 years	8.729	0.445	171.224	0.154
≥10 years	0.625	0.156	2.503	0.507

HIE, Healthy immigrant effect; AOR, adjusted odds rate; HIE, healthy immigration effect; CI, confidence interval.

### 3.4. Model validation

**Figure 2:**
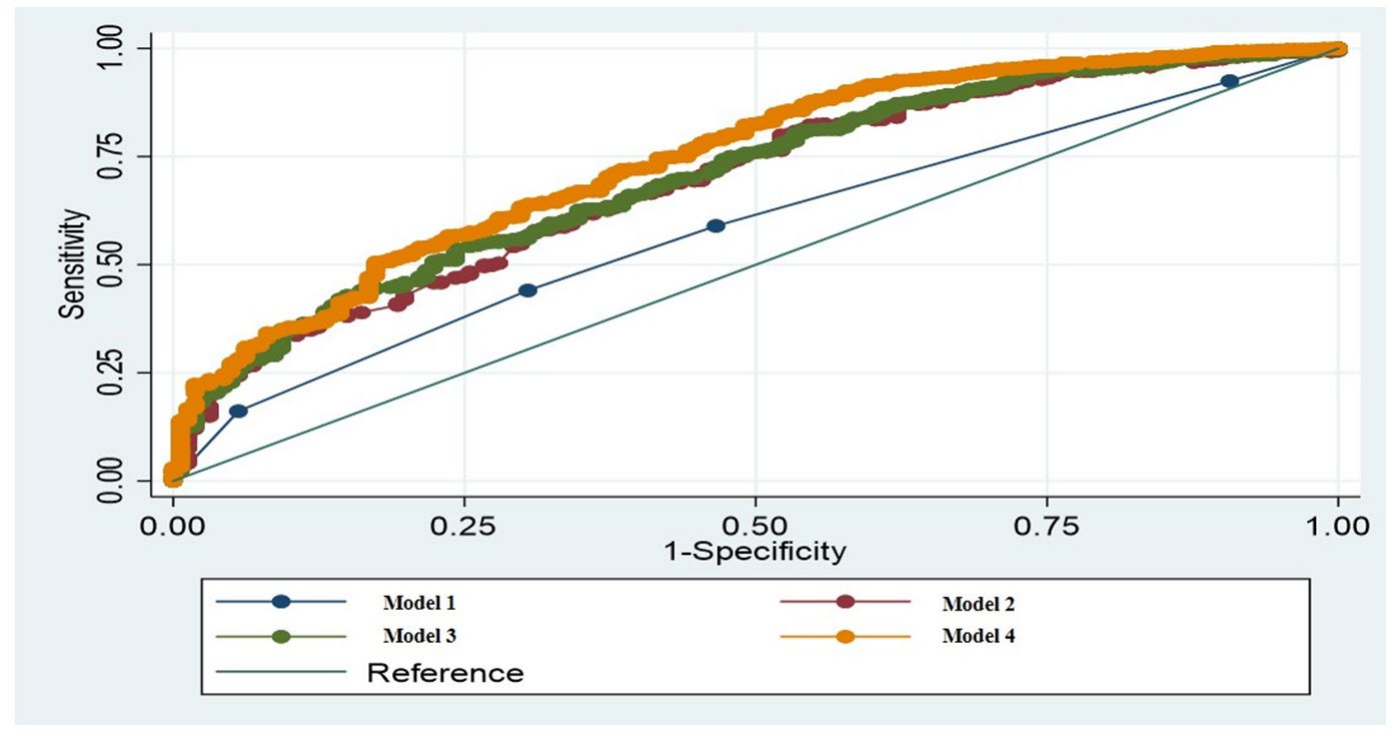
Comparisons of ROCs for models 1, 2, 3, and 4. ROC, receiver operating characteristic.

According to the validation results, model 4 had the highest AUC (0.7431), followed by model 3 (AUC = 0.7070), model 2 (AUC = 0.6948), and model 1 (AUC = 0.5891). Further, data fitting revealed that the Akaike information criterions of models 1, 2, 3, and 4 were 929.285, 844.247, 846.8031, and 814.554, respectively (Figure 2). Therefore, model 4 was selected as the final model.

## 4. Discussion

This study analyzed the HIE theory for internal migrants in a megacity and found that migrants had a health advantage over native locals. However, this health advantage did not apply to those who had lived in Shanghai for >10 years. Additionally, marital status, educated with a graduate degree or above, an annual income of CNY 110,000–250,000, number of physical examinations undergone in the past 12 months, and number of critical diseases they were suffering from, had significant effects on SRH.

The results of this study showed that when confounding variables were controlled, internal migrants who had lived in Shanghai for 5–10 years were 2.418 times more likely to report good health than Shanghai locals; this finding supported the HIE theory and was consistent with that of other studies (25–29). For example, Kwak (30) argued that compared with native-born Canadian adolescents, Canadian immigrant adolescents exhibited a significant HIE regarding their physical and mental health; in other words, they were healthier than the native-born adolescents. These findings may be explained by the self-selection effect or the cultural buffering effect. Many scholars have suggested that migrants are “self-selected” in their country of origin and that the healthiest individuals are “selected” to migrate (31–33). Such migrants may be more educated, less frequently exposed to health risks, and more entrepreneurial, making them more likely to migrate (34). Consequently, the health of migrants is often favorable to that of locals when migrants first arrive in their destination country. Cultural buffering refers to a protective buffering effect that stems from a migrant’s culture and lifestyle. Migrants from countries in the Global South tend to maintain healthier lifestyles (such as abstaining from drugs and alcohol and managing body weight more effectively) than the native people of the destination countries (35). According to Wilkinson et al. (14), the years of residency for migrants could be classified into the following three stages: short-term, medium-term, and long-term; with the stage of 5–10 years corresponding to the medium-term. By this time, internal migrants may be more confident in their new language (although perhaps not fluent) and more familiar with the new culture and lifestyle. Conversely, the short-term (<5 years) migrants may still be learning a new language and culture, and adapting to a new environment. Compared with short-term migrants, the medium-term migrants, with more self-esteem and more mastery, are expected to benefit more from the aforementioned self-selection effect or the cultural buffering effect, and encounter less health problems. This finding was in line with that of previous studies (3, 36). Moreover, migrants’ social networks upon arrival can further reinforce positive health behaviors and provide psychological support (37). The communities in which migrants live can also provide resilience against psychological pressures (38, 39). Therefore, to maintain the health advantages of migrants, the government should take measures to intervene, such as improving physical examinations and screening for internal migrants, promoting healthy lifestyles and behaviors (e.g., tobacco abstinence), and establishing resilient community protection mechanisms.

Further, in this study, the OR of SRH for internal migrants who had lived in Shanghai for >10 years was not significant; this finding suggests that the health advantages of the HIE among internal migrants gradually decline over time. This decline may be caused by acculturation and the persistent effect of the local environment as well as a cumulative exposure to it (40, 41). The persistent effect of the local environment refers to the decline in health advantages among migrants as they continue to live in their host country. Acculturation refers to the assimilation of lifestyle and language of the host country or region. Migrants tend to adopt the lifestyle of locals and gradually abandon their original lifestyle. Studies have shown that when migrants reside for longer periods in their host country, they tend to gradually assimilate, and their health tends to decline to the level reported by the local population (42). Cumulative exposure refers to the time spent experiencing stressors at multiple levels, such as the individual level (for example, financial constraints and language barriers), socioeconomic level (for example, discrimination, racism, and unequal job opportunities), and organizational level (for example, food monitoring, housing, and suffering because of poor health-care and social care systems) (43). These stressors gradually worsen over time and eventually have a significant effect on a person’s health (44). Therefore, with the increase in the length of stay of migrants in Shanghai, some acculturative factors, such as their language, diet, social interactions, and customs, gradually integrate with the local lifestyle. Moreover, internal migrants face more competition and economic pressure, which may lead to a gradual decline in mental health and the loss of health advantages. Overall, in this study, through the mechanism of HIE, internal migrants exhibited more favorable SRH status than Shanghainese, most likely due to the self-selection and the cultural buffering effect, as well as the social network support provided by the communities in which they settled. However, the health advantage disappeared when the length of residence exceeded a certain level and was affected by cumulative exposure experienced in the destination of migration.

Therefore, the government should enact measures that consider the length of residency, for example, by attaching more importance to number of years of residency and employment in the point-based hukou household registration system, promoting the urbanization of migrants, and providing more public services and social security to permanent residents of Shanghai. Moreover, popularizing the Shanghainese dialect among migrants, promoting the integration of their hometown dialects with the local dialect, and providing regular mental health counseling are also necessary.

Furthermore, other covariates were significantly associated with SRH among internal migrants, including being married, having a graduate degree or above, having an annual income between CNY 110,000 and CNY 250,000, receiving more than one physical examination in a year, and having one or more critical diseases. These findings are consistent with those of other studies regarding SRH (20, 21, 23, 24, 45, 46). A higher physical examination frequency among migrants can indicate greater concerns over health and healthy behaviors (44). However, some scholars have suggested that the frequency of physical examinations is a consequence of SRH and is complicated by other determinants, such as availability of health-care resources (46–48). Migrants with an annual income between CNY 110,000 and CNY 250,000 reported a better health status than those with an annual income <CNY 100,000. This finding indicates that having a higher income improves the living standards of internal migrants and gradually improves their SRH. Improvements to living standards can in turn reduce the burden of medical expenses and facilitate access to treatment. However, this was not the case for those with an annual income >CNY 250,000, whose OR of SRH was not significant. Therefore, internal migrants who have a higher income are not necessarily healthier; this finding, which is consistent with those of other studies (21, 49, 50), can be explained by the marginal effect of subjective well-being. Specifically, as their income increases, internal migrants may face greater pressure and competition, which can negatively affect their subjective well-being and result in a decline in SRH (50). Moreover, the relationship between SRH and income may be confounded by socioeconomic factors such as employment status, social support, family structure, and time trends, and is, therefore, currently inconclusive (21). Among the internal migrants analyzed in this study, those who had one or more critical diseases were less likely to report being healthy than those with no critical diseases, possibly because serious diseases can directly affect one’s own perceived physical and mental health status and lead to poor SRH (23, 24). However, some studies have suggested that SRH affects the incidence of chronic diseases (51, 52). Therefore, the government should seek to understand the associations of individual characteristics, socioeconomic status, health-care use, and chronic diseases with the SRH of internal migrants and enact measures to (i) improve such migrants’ socioeconomic conditions, (ii) increase their health-care use (for example, offer routine physical examinations and convenient medical treatment), (iii) improve the screening technology for identifying critical diseases, and (iv) expand health education communication that targets internal migrants.

Finally, according to the findings of this study, the HIE is not applicable to white-collar migrants. White-collar workers in megacities like Shanghai may face more workplace competition and daily stress compared with workers in other industries, which ultimately affects their physical and mental health. According to a report published in 2020, 94% of a sample of white-collar workers suffered from chronic diseases (53). A white-collar worker has 5.4 different health problems on average, and subpar health has become common among such workers. Unhealthy lifestyle traits and behaviors, such as engaging in sedentary work, keeping late hours, having an irregular diet pattern, experiencing high stress levels, and having poor sleep quality, are the root causes of many health problems among white-collar workers, resulting in the absence of health benefits from HIE, regardless of their length of residency in the migration destination. By contrast, in this study, blue-collar workers in the manufacturing industry reported relatively favorable health, especially those who had lived in Shanghai for 5–10 years; this finding supports the HIE and might have resulted from the fact that China has a relatively mature legal system for protecting the health of blue-collar workers and provides benefits such as early retirement. By contrast, no such benefits are granted to white-collar workers (54, 55). Therefore, the government should enact laws and policies to increase protection for white-collar migrants both inside and outside the workplace and to efficiently identify work-related health conditions. Moreover, the government could enact measures that promote health education, address sedentary working conditions, improve health management (for example, free physical examinations and cancer screenings), make physical exercise options more accessible, and improve physical and mental health among internal migrants (56–58).

This study had some limitations. Firstly, data for the study were collected from a specific manufacturing industry; thus, the results might not be extendable to other industries. Secondly, only SRH was used to verify the HIE; although SRH is undeniably a well-validated health measure (51) that has been used and validated in multiple settings including China (59), subjective health assessment, which was used to capture health status in this study, could have led to inevitable response bias and reverse causation. Therefore, in future studies, other objective indicators (such as mortality and mental health) should be considered to measure the health status. Additionally, some key variables should be considered, such as intergenerational families, age, and family structure. Thirdly, a cohort effect might have been caused by other factors related to the duration of residency, which in turn might have led to homogeneity or heterogeneity in the SRH among the different groups of internal migrants. Fourthly, because health might have a reverse causal relationship with migration, leading to an endogeneity problem, an instrumental variable (IV) should be introduced into the logistic regression. Therefore, the use of an appropriate IV for internal migration status, such as the share of migrating households (60), migrant network (61), or sibling age (62), is needed as a robustness test to mitigate the endogeneity problem in the model. Finally, self-reported variables might have been influenced by a recall bias; for example, an individual’s SRH status might have fluctuated over time as well as according to individual demographic or socioeconomic characteristics.

In conclusion, this study analyzed the HIE theory and applied it to evaluate SRH. Compared with Shanghai locals, internal migrants initially had more favorable SRH; however, these health advantages gradually diminished as the duration of residency increased. Thus, the Chinese government should carefully consider the heterogeneity of SRH among internal migrants and their length of residency to formulate appropriate policies and health promotion programs. In particular, the government should factor in the less favorable SRH of migrants who have lived in Shanghai for >10 years. Improvements to physical screening technology, promotion of healthy habits, and facilitation of integration should also be considered. Finally, some community-based psychological interventions may be necessary to address mental health, alleviate cumulative exposure to the local environment, and improve socioeconomic and working conditions for internal migrants, especially white-collar workers.

## Data availability statement

The raw data supporting the conclusions of this article will be made available by the authors, without undue reservation.

## Ethics statement

The studies involving human participants were reviewed and approved by Shanghai University of Medicine and Health Sciences Institutional Review Board for the Protection of Human Subjects. The patients/participants provided their written informed consent to participate in this study.

## Author contributions

ED, TX, ZL, and CH designed the study and acquired the data and developed the statistical plan. JS and DB carried out the survey. ED and TX performed the statistical analysis. ED, TX, ZL, HZ, and CH interpreted the analysis. ED, TX, ZL, and CH drafted and revised the manuscript. All authors contributed to the article and approved the submitted version.

## Funding

This research was funded by National Social Science Foundation of China General Project (Grant No. 19BGL246) and National Social Science Foundation of China Major Project (Grant No. 18ZDA088).

## Conflict of interest

The authors declare that the research was conducted in the absence of any commercial or financial relationships that could be construed as a potential conflict of interest.

## Publisher’s note

All claims expressed in this article are solely those of the authors and do not necessarily represent those of their affiliated organizations, or those of the publisher, the editors and the reviewers. Any product that may be evaluated in this article, or claim that may be made by its manufacturer, is not guaranteed or endorsed by the publisher.

## Abbreviations

HIE: Healthy immigrant effect
SRH: Self-rated health
