# Supplementary material for: Healthy immigration effect among internal migrants in megacities: a cross-sectional study in Shanghai, China
Source: Front Public Health. 2023 Jun 12;11:1167697. doi: 10.3389/fpubh.2023.1167697 (PMC10291071; doi:10.3389/fpubh.2023.1167697)
Supplement: Supplementary file 1 [file Data_Sheet_1.PDF]

Supplementary Table 1. Comparison of demographic profile of internal migrants between the study sample and official data of Shanghai's internal migrant population

	The sample of internal migrants in the study (n=1024)	Shanghai's Internal migrant population from official data in 2020[a] (N=10,467,000)
Characteristics		
**Age**		
≤39 old years	864（84.4%）	7,250,491(69.27%)
40-60 old years	160（15.6%）	2,528,827(24.16%)
**Gender**		
Female	475（46.4%）	4,961,358(47.4%)
Male	549（53.6%）	5,505,642(52.6%)
**Marital status**		
No	206（20.1%）	2,449,278(23.4%)
Yes	818（79.9%）	8,017,722(76.6%)
**Educational level**		
High school or below	53（5.2%）	7,264,098 (69.4%)
University (including junior college)	827（80.8%）	2,847,024 (27.2%)
Graduate or above	144（14.0%）	355,878 (3.4%)
**Years of residency**		
**<1 year**	359（35.1%）	1,256,040 (12.0%)
1-5 years	142（13.9%）	4,040,262 (38.6%)
≥5-10 years	523（51.0%）	5,170,698 (49.4%)

a:data source: Xia G, Zhu Y, Lin L, Ke W. Migrants' multidimensional integration in cities and regional differences in the three major economic regions of China's eastern coastal area. Prog. Geogr. 2018;37: 373-84. HIE: Healthy immigrant effect
